# Evolution of Percutaneous Nephrolithotomy (PCNL) from Standard to Miniaturized and Ultra-Mini Techniques: A Narrative Review

**DOI:** 10.3390/medicina62030484

**Published:** 2026-03-04

**Authors:** Mladen Doykov, Jasmin Gurung, Usman Khalid, Gancho Kostov, Bozhidar Hristov, Petar Uchikov, Krasimir Kraev, Lyubomir Chervenkov, Elizabet Karen Dzhambazova

**Affiliations:** 1 Department of Urology and General Medicine, Medical Faculty, Medical University of Plovdiv, 4002 Plovdiv, Bulgaria; gancho.kostov@mu-plovdiv.bg; 2 Medical Faculty, Medical University of Plovdiv, 4002 Plovdiv, Bulgaria; jasmingurung12@gmail.com (J.G.); usmankhalid957@gmail.com (U.K.); 3 Second Department of Internal Diseases, Section “Gastroenterology”, Medical Faculty, Medical University of Plovdiv, 4002 Plovdiv, Bulgaria; bozhidar.hristov@mu-plovdiv.bg; 4 Department of Special Surgery, Faculty of Medicine, Medical University of Plovdiv, 4002 Plovdiv, Bulgaria; petar.uchikov@mu-plovdiv.bg; 5 Department of Propedeutics of Internal Diseases, Medical Faculty, Medical University of Plovdiv, 4002 Plovdiv, Bulgaria; krasimir.kraev@mu-plovdiv.bg; 6 Department of Diagnostic Imaging, Medical Faculty, Medical University of Plovdiv, 4002 Plovdiv, Bulgaria; lyubomir.chervenkov@mu-plovdiv.bg; 7 Department of Social Medicine and Public Health, Public Health Faculty, Medical University of Plovdiv, 4002 Plovdiv, Bulgaria; betidzhambazova@gmail.com

**Keywords:** percutaneous nephrolithotomy, miniaturized PCNL, mini-PCNL, ultra-mini PCNL, micro-PCNL, nephrolithiasis, stone-free rate, intrarenal pressure, vacuum-assisted PCNL

## Abstract

*Background and Objectives:* Because of its consistently high stone-free rates (SFRs), percutaneous nephrolithotomy (PCNL) continues to be the first-line treatment for renal stones larger than 20 mm. Standard 24 to 30 Fr access tracts, however, are linked to access-related morbidity, such as bleeding, pain, and extended hospital stays. These restrictions have led to progressive tract miniaturization and the development of mini-PCNL, ultra-mini PCNL, and micro-PCN techniques. *Materials and Methods:* We performed a narrative review of studies published through January 2026 using PubMed and Google Scholar. Search terms included percutaneous nephrolithotomy, mini-PCNL, ultra-mini PCNL, micro-PCNL, and vacuum-assisted PCNL. Original studies, systematic reviews, and meta-analyses reporting clinical outcomes, complications, and advancements were selected, whereas conference abstracts, non-English papers, and articles without accessible full text were excluded. *Results:* Across randomized trials, miniaturized PCNL generally preserves efficacy when patients are selected appropriately. Across randomized trials and meta-analyses, miniaturized PCNL achieved stone-free rates comparable to standard PCNL (typically ~80–90% for stones ≤20 mm and similar rates in selected stones >2 cm), while demonstrating lower hemoglobin decrease (mean difference approximately −0.6 to −1.0 g/dL), reduced transfusion rates, and shorter hospital stays, at the cost of longer operative time (mean difference ~8–12 min). On the other hand, operative time may increase, and smaller working channels can make visualization and fragment evacuation more demanding as stone burden rises. Raised intrarenal pressure is a recurring safety issue because it may increase infectious risk unless drainage is actively managed. Recent innovations aim to address these limitations, including vacuum-assisted access sheaths, pressure-controlled irrigation, improved laser and lithotripsy platforms, image-fusion guidance, navigation systems, and robotic assistance. *Conclusions:* PCNL now spans a spectrum of tract sizes rather than a single standard approach. When chosen appropriately and performed with attention to pressure control and fragment evacuation, miniaturized PCNL can reduce morbidity without sacrificing stone clearance. Future advancements in percutaneous stone surgery are more likely to rely on integrated technological solutions that improve accuracy, safety, and repeatability than on additional tract size reduction.

## 1. Introduction

Approximately one-third of the surgical workload in the urological department is associated with the treatment of renal and ureteral stones [1]. Nephrolithiasis represents a growing global health burden, with an increase in incidence and prevalence related to climate, dietary, and socioeconomic factors [2]. According to international guidelines, percutaneous nephrolithotomy (PCNL) is the first-line treatment for stones larger than 20 mm because of its high stone-free rate (SFR). Stones measuring 10 to 20 mm can be managed by shock wave lithotripsy (SWL), PCNL, or retrograde intrarenal surgery (RIRS) [3].

Prior to the advent of less invasive techniques, most symptomatic patients with renal calculi were treated with surgical lithotomy [4]. PCNL was first introduced in 1976; however, as other noninvasive, easy, and effective treatments like shock wave lithotripsy gained popularity in the early 1980s, the use of PCNL diminished [5,6].

Recent interest in this field has been reignited due to two important factors. Growing clinical experience has led to a significant awareness of the limitations of shock wave lithotripsy [7]. A second key factor was the improvements in PCNL techniques, leading to decreased morbidity and stone-free rate (SFR) of more than 90% in treated patients [8].

Even though standard PCNL achieves excellent SFRs in the management of large, multiple, or complex renal stones, it is associated with greater morbidity, including postoperative pain, transfusion rates, hemoglobin drop, and longer hospital stay. This balance between maximum SFR and minimum patient harm has been a major catalyst for the innovation of miniaturized PCNL techniques [9,10].

To minimize the invasiveness, especially for children, Jackman et al. introduced the first mini-percutaneous nephrolithotomy (mini-PCNL) technique. Smaller access sheaths were used, demonstrating the feasibility of tract miniaturization [11]. This concept was subsequently adapted for adults when Lahme et al. pioneered the first adult mini-PCNL [12]. Since its inception, technological advances have led to many modifications and refinements in techniques and the instrument itself. The introduction of miniaturization of instruments has led to the creation of ‘mini-PCNL,’ ‘ultra-mini PCNL,’ and ‘micro-PCNL.’

These techniques aimed to maximize the stone-free rate (SFR) while minimizing patient harm and reducing the morbidity associated with standard PCNL [13].

Despite the undeniable advances, important clinical questions remain unanswered. For example, it is unclear whether miniaturized PCNL techniques can consistently maintain stone-free efficacy across clinically relevant stone burdens. Additionally, it is questionable whether reductions in tract size result in trade-offs in operative efficiency, intrarenal pressure control, and infection risk. At the same time, technological advances such as suction-enabled access sheaths, pressure-modulating systems, and image-guided or navigated platforms have emerged as potential strategies with the aim of addressing the technical constraints imposed by miniaturization.

This narrative review aimed to critically examine the evolution of PCNL from standard to miniaturized techniques, to compare clinical outcomes, to define the balance between efficacy and safety, and to assess the extent to which emerging technologies can compensate for the constraints imposed by smaller access tracts.

## 2. Materials and Methods

This article presents a narrative literature review of the evolution of percutaneous nephrolithotomy from standard PCNL to miniaturized and ultra-mini techniques. The rationale for opting for a narrative review rather than a full systematic review of meta-analysis was prompted by the need to integrate clinical outcomes with technical and mechanistic aspects that are not appropriate for quantitative pooling. Furthermore, the considerable heterogeneity in outcome definitions and the rapidly changing PCNL platforms and technologies led to that decision. We conducted a search of the Google Scholar and PubMed databases for all relevant publications from early to recent, up until January 2026. The search terms included “percutaneous nephrolithotomy,” “mini-PCNL,” “ultra-mini PCNL,” “micro-PCNL,” and “vacuum-assisted PCNL.” We also screened the reference lists of included articles for other relevant studies.

### 2.1. Eligibility Criteria

The authors independently screened abstracts and titles before conducting a full-text review for relevance, with disagreements being settled by consensus. Eligible studies included English-language original research articles, systematic reviews, and meta-analyses addressing standard PCNL and/or miniaturized PCNL techniques (including ultra-mini and micro-PCNL). We included comparative studies, randomized controlled trials, and cohort studies. Studies were eligible if they reported at least one of the following: clinical outcomes, perioperative metrics, complications, or technological/technical aspects of PCNL (including miniaturized instrumentation and suction or vacuum-assisted systems). We excluded non-English publications, conference abstracts, and articles for which the full text was not accessible. The rationale for excluding non-English publications was that studies in other languages are not often available in full text. Furthermore, professional translations of such texts are not always possible, and selecting only the available ones could introduce another bias. Nevertheless, we recognize that excluding potentially relevant studies published in other languages presents a limitation to the scope of this review.

### 2.2. Qualitative Synthesis

First, we focused on standard PCNL and its limitations, which subsequently led to its miniaturization. We then summarized the evolution of miniaturized PCNL platforms, describing mini-PCNL, ultra-mini PCNL, and micro-PCNL with attention to how progressive reductions in tract size and instrument caliber aimed to maintain effectiveness while reducing morbidity. Next, we organized the literature around key comparative domains that are consistently reported across studies.

**Figure 1:**
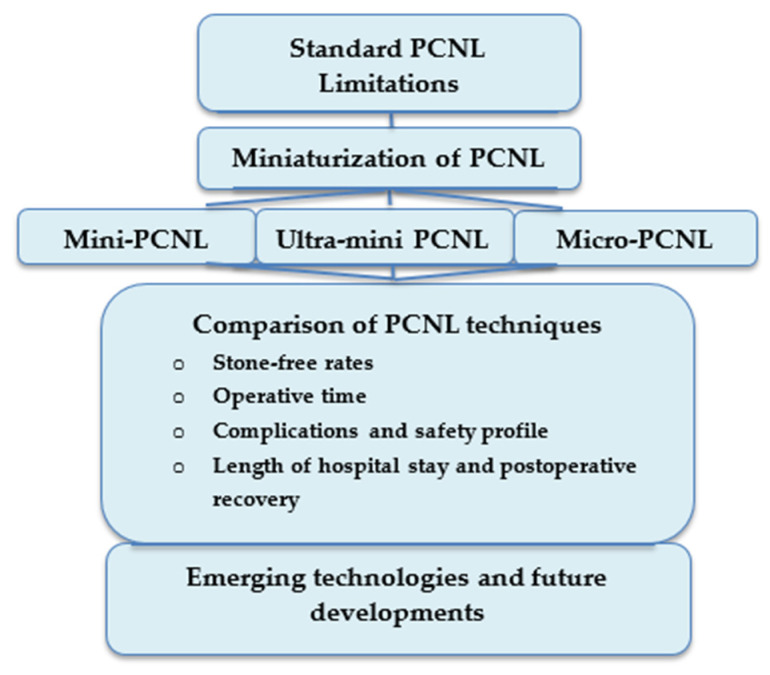
Conceptual overview of the evolution of PCNL and the outcomes assessed in this narrative review.

Stone-free rates (SFRs), perioperative and postoperative complications, operative time, postoperative recovery metrics, and length of hospital stay were among these domains. Lastly, we compiled information on new technologies and method improvements that could further impact miniaturized PCNL, such as pressure-controlled irrigation, suction or vacuum-assisted access sheaths, and other workflow innovations intended to enhance visibility, ease fragment clearance, lower intrarenal pressure, and possibly lessen infectious complications (Figure 1).

## 3. From Standard to Miniaturized PCNL: Techniques, Outcomes, and Emerging Innovations

### 3.1. Development of Standard PCNL

The introduction of PCNL provided a foundation for surgical evolution within urology. The first case report for its stone-based use under radiological control dates to 1976 and highlights the start of a transition from open surgery dominance. It serves as a historical point of reference for percutaneous access to the collection system, despite being mainly a proof-of-concept [5]. Early PCNL was devised in two main stages, including access and drainage, followed by stone removal. However, a key shift in the procedural workflow was highlighted in Wickham et al.’s research, which showcased a single-stage PCNL with a simplified procedure and reduced postoperative hospital stays [14]. Typical tract sizes vary from 24 to 30 Fr, permitting the utilization of rigid nephroscopy and effective intracorporeal lithotripsy without compromising visibility and operative efficiency. Standard PCNL became the preferred intervention for patients with large renal calculi exceeding 2 cm, staghorn stones, and complex stone burdens that were unsuitable for shock wave lithotripsy or ureteroscopy. Optimized stone-free rates and reproducible multi-center outcomes led to its acceptance as a definitive measure for large renal calculi. Currently, it is considered the gold standard approach for complex nephrolithiases [15]. To ensure its effectiveness and in routine clinical practice, results must be reproducible. A CROES global study, involving 5724 PCNL patients treated with PCNL at different centers around the world, reported high stone-free rates across a wide range of stone sizes and complexities. These outcomes were observed in various healthcare environments, demonstrating PCNL’s broad applicability. Its effectiveness in complex cases, including staghorn calculi, contributed to its continued dominance as the primary surgical option for large renal stones [16].

### 3.2. Limitations of Standard PCNL

Despite the benefits of standard PCNL, it has been associated with clinical morbidity and complications. In two global CROES studies, one already mentioned in Section 3.1 with 5724 patients [16] and another involving 5803 patients, at 96 centers around the world [17], complications were evaluated following the modified Clavien classification, which includes five grades, determined by to the severity of the adverse events and the type of treatment needed to address them. In the first global CROES study, 20.5% of the patients experienced at least one complication, with bleeding and postoperative fever—temperature reaching above 38.5 °C—being the most common adverse events. Two postoperative deaths indicated a variability in the success rate of PCNL. Furthermore, patients with physical status III and IV according to the American Society of Anesthesiologists (ASA), as well as those with anticoagulant use, positive urine culture, and concurrent cardiovascular disease, experienced a higher complication burden. ASA status and operative time were identified as significant predictors of higher complication grades [16]. In the second global CROES study, procedure-related complications included significant bleeding (7.8%), renal pelvis perforation (3.4%), and hydrothorax (1.8%). Blood transfusion was required in 5.7% of cases, and postoperative fever with temperature reaching above 38.5 °C occurred in 10.5% [17]. The distribution of complications according to the modified Clavien classification was as follows: 11.1% of complications were classified as Clavien I, 5.3% as Clavien II, 2.3% as Clavien IIIa, 1.3% as Clavien IIIb, 0.3% as Clavien IVa, 0.2% as Clavien IVb, and 0.03% as Clavien V [17].

Large cohort studies link bleeding risk to access-related factors intrinsic to standard-caliber tracts. Compared with telescopic dilation, balloon tract dilation was associated with longer median operative time (94 vs. 60 min) and higher rates of bleeding (9.4% vs. 6.7%) and transfusion (7.0% vs. 4.9%). In 5537 PCNL cases, sheath size was an independent predictor of bleeding, as it directly impacted tract caliber in hemorrhagic morbidity [18]. Tract size, multiple access tracts, operative time, diabetes mellitus, and intraoperative complications were identified as significant predictors of blood loss in a prospective study with 236 patients undergoing 301 PCNL procedures. The overall transfusion rate was 7.9% [19]. Review articles concentrating on complications have similarly highlighted that, although less invasive than open surgery, PCNL possesses a unique and procedure-specific morbidity profile that must be considered alongside its high efficacy [20].

### 3.3. Rationale for Miniaturization of PCNL

The primary goal of PCNL’s miniaturization was to reduce access-related morbidity while preserving high stone clearance, which is a major advantage of this kind of surgery. Large-scale studies indicate that while most standard PCNL procedures are uneventful, clinically important complications—such as bleeding and the need for transfusions—are still associated with creating and working through a large renal tract. The complications observed in both global CROES studies [16,17] provide a rationale for miniaturization. If a substantial fraction of morbidity is tract-driven, then reducing the tract diameter should decrease parenchymal trauma and vascular injury risk. This method has the potential to lower hemoglobin drops and the need for transfusions without abandoning percutaneous access.

Miniaturization was not adopted solely on theoretical grounds. It became credible when comparative outcome data suggested that smaller tracts can maintain efficacy (stone-free outcomes) while improving bleeding-related endpoints. A meta-analysis including only randomized controlled trial studies reported that miniaturized PCNL (≤22 Fr) was associated with lower transfusion rates (odds ratio = 0.33, 95% CI: 0.17–0.63) and a smaller hemoglobin drop (mean difference = 0.72) compared with standard PCNL, but at the cost of longer operative time (mean difference = 10.98 min). They also noted that for stones ≥ 2 cm, the difference in stone-free rate between groups was not significant, indicating that miniaturized approaches can be applied beyond small stone sizes if technique, fragmentation strategy, and multi-tract approaches are optimized [21]. Another meta-analysis comparing mini-PCNL versus standard PCNL, reported non-inferior stone-free rates (odds ratio = 1.10), alongside smaller hemoglobin decrease (mean difference = 0.68), fewer transfusions (odds ratio = 0.36), and shorter inpatient stays (mean difference = 0.81 days). Overall adverse event differences were not always statistically robust across heterogeneous definitions and techniques [22]. Both meta-analyses concluded that the main benefits of miniaturized PCNL were related to hemorrhage and recovery. On the other hand, technical complexity presented a trade-off but with no influence on its efficacy.

Examining earlier and later “mini” platforms as intentional reactions to the same morbidity drivers helps to clarify the clinical justification. By reducing the working tract while maintaining a percutaneous approach, Jackman et al. clearly framed the original “mini-perc” (13-Fr) as an attempt to address the shortcomings of standard PCNL [23]. Subsequent improvements used developments in lithotripsy and endoscopy to make miniaturization technically feasible at scale. Smaller access with effective dusting, as opposed to larger access with fragment extraction, was the design philosophy behind a modified ultra-mini technique that used a mini-nephroscope through an 11–13 Fr metal sheath with holmium YAG lithotripsy for stones smaller than 20 mm [24]. Microperc marked a further advancement. It was defined as a single-step access-and-treatment strategy using a 4.85 Fr needle, explicitly targeting further reductions in tract-related morbidity by minimizing dilation. While these descriptions are not comparative efficacy proofs by themselves, they are important because they show miniaturization evolving as a morbidity-mitigation engineering problem [25].

Evidence also supports the rationale at the patient level where standard PCNL morbidity is most consequential. A randomized clinical trial compared ultra-mini PCNL and standard PCNL for 10–20 mm renal or upper ureteric stones, with clearly distinct access calibers (16-Fr sheath with a 9.8-Fr ureteroscope vs. 30-Fr sheath with a 24-Fr nephroscope). There were statistically significant advantages for ultra-mini PCNL in postoperative hemoglobin levels, transfusion rates, hospitalization duration, and VAS pain scores, while no significant differences in stone-free rate or operation time were observed. This highlights how downsizing the tract can translate into clinically meaningful reductions in bleeding and recovery burden without sacrificing stone-free rate [26].

Miniaturization has its own physiologic and technical risks, which present a crucial caution factor. This is part of the reason why there are several “mini” variants rather than a single widely used technique. Reduced outflow through smaller sheaths can raise intrarenal pressures, which is linked to infectious complications. Higher renal pelvic pressure with reduced tract diameter and irrigation outflow are considered key determinants [22]. Meanwhile, smaller-caliber access can reduce visual field, slow fragment evacuation, and increase operative time, which are all signals that appear consistently in comparative literature [21]. Therefore, the rationale for miniaturization is not simply to reduce size but to address tract-related morbidity inherent to standard PCNL. Since many complications are related to access size, systematic reduction in tract caliber is rational. Recent advances in optics, laser technology, and fragmentation techniques enable effective stone treatment through smaller tracts. This requires careful management of intrarenal pressure, visualization, fragment management, and appropriate case selection.

### 3.4. Miniaturized PCNL Techniques

#### 3.4.1. Mini-PCNL

Mini-PCNL is a type of percutaneous renal stone surgery that is performed through a reduced-caliber working tract, usually in the mini-tract range (between 14 and 22 Fr). The goal is to keep the benefits of direct calyceal access and endoscopic lithotripsy while reducing the risk of parenchymal trauma from access. Miniaturized PCNL has been variably defined across platforms and studies, with substantial heterogeneity in tract size, instrumentation, and operative technique reported in the literature, complicating direct comparison between published series [27].

The procedural framework broadly mirrors standard PCNL through image-guided calyceal puncture, guidewire placement, tract dilation, endoscopic lithotripsy, and drainage decisions. Despite this, mini-PCNL fundamentally changes fluid mechanics and fragment logistics within the collecting system. Using a 14 Fr peel-away sheath, the early adult “mini-perc” demonstrated the viability of operating through a smaller tract and brought attention to the practical limitations that still exist today. These include limited extraction capacity and decreased passive outflow, as well as a shift in procedural efficiency away from rapid evacuation of large fragments and toward controlled clearance and fragmentation [28]. Contemporary mini-PCNL therefore places greater emphasis on aligning the energy source and fragmentation strategy with a constrained working channel and on selecting adjuncts—such as baskets, irrigation-assisted washout, or aspiration-enabled systems—to avoid prolonged manipulation and repeated scope exchanges [29].

A key technical and safety determinant in mini-PCNL is intrarenal or intrapelvic pressure (IRP/IPP), which is intrinsically influenced by sheath caliber, irrigation settings, and outflow. Multiple experimental studies demonstrate that miniaturization tends to increase pressure compared with standard tracts, reinforcing the need for pressure-aware techniques rather than assuming smaller access is universally safer. In a porcine model designed to simulate an infected collecting system, the use of a 14/16 F ureteral access sheath versus a standard 30 F sheath produced higher mean intrapelvic pressures (18.76 ± 5.82 mmHg vs. 13.56 ± 5.82 mmHg). The time spent above 30 mmHg was 117.0 vs. 66.1 s. Higher rates of end-organ bacterial seeding were observed, thus showing evidence that elevated pressures during mini-PCNL may increase the potential for complications in an infected system [30].

The pressure dynamics can be reproduced across different models and device configurations. A silicone urinary tract model compared flexible ureteroscopy, mini-PCNL, standard PCNL, and endoscopic combined intrarenal surgery (ECIRS). Intrapelvic pressure (IPP) during mini-PCNL ranged between 2.4 and 39.7 cmH_2_O, whereas standard PCNL pressures were lower. Lower irrigation pressure (40 cmH_2_O vs. 193 cmH_2_O) and larger mini operating sheaths (>15/16 Fr) were associated with reduced IPP [31]. In an ex vivo porcine urinary tract model, pressures during mini-PCNL were significantly higher than during conventional PCNL across tested variations. Modifiable factors such as reduced irrigation height and ureteric catheterization significantly lowered the intrarenal pressure (IRP) [32]. These findings show that mini-PCNL requires deliberate control of irrigation and outflow to prevent pressure-related risk. In some situations, downsizing without pressure mitigation may compromise infectious vulnerability for bleeding reduction.

Instrument and sheath innovations have therefore become central to modern mini-PCNL, particularly systems designed to improve fragment clearance while stabilizing pressure. Vacuum-assisted mini-PCNL platforms represent an attempt to address two mini-PCNL limitations simultaneously: limited fragment egress and elevated intrarenal pressure. In a prospective series describing vacuum-assisted mini-PCNL performed via a 16Ch ClearPetra sheath, intrarenal pressure monitoring demonstrated that mean intrarenal pressure (IRP) remained below the threshold. It was associated with pyelovenous backflow for most procedures, with only brief cumulative time above that limit. The authors also identified procedural steps during which IRP may rise, reinforcing the importance of workflow-specific pressure control [33]. Broader evaluations of aspiration-assisted sheaths also characterize suction-enabled designs as a method to improve evacuation and reduce pressure excursions. However, they also emphasize heterogeneity in device configuration and reporting standards, which restricts direct comparability across different platforms [34].

Mini-PCNL and standard PCNL are not interchangeable from a practical perspective. Each situation should be considered individually when selecting a mini-PCNL. The technical advantages are most compelling when access-related morbidity is a priority, while operative efficiency depends on matching stone characteristics (burden, hardness, location) to the chosen fragmentation and evacuation strategy. Additionally, it is important to consider pressure-mitigation techniques such as regulated irrigation, adequate outflow, decompression methods, and suction-enabled systems when necessary. As a whole, mini-PCNL is a technically advanced percutaneous variation with a safety-efficiency profile that is extremely dependent on evacuation mechanics and pressure control. Its implementation should specifically address these limitations [27,29].

#### 3.4.2. Ultra-Mini PCNL

Ultra-mini-PCNL (UM-PCNL) was designed to reduce the tract size even further than mini-PCNL, featuring a 1 mm (3 F) telescope in conjunction with a 7.5 F nephroscope. The miniaturization reduced the cross-sectional surface area to nearly one-eighth of the original tract size in comparison to conventional PCNL. This modification was intended to decrease bleeding and tissue trauma, ultimately further reducing morbidity [35]. In a randomized clinical trial, UM-PCNL presented comparable stone-free rates to standard PCNL for renal and upper ureteric stones of a diameter less than 20 mm. The UM-PCNL cohort experienced notable reductions in perioperative morbidity with significantly smaller hemoglobin drops, shorter hospital stays, reduced postoperative pain scores, and analgesic requirements. The transfusion rates were also significantly lower compared to standard PCNL without the requirements for further interventions [26].

In addition to comparative trials, prospective case series support the safety and efficacy of UM-PCNL for managing small renal stones. One hundred and twenty patients underwent UM-PCNL for unilateral renal stones ranging from 8 to 20 mm, and it resulted in a stone-free rate higher than 99% at the two-week follow-up. The initial fragmentation was successful in 95% of cases with no significant postoperative complications. Only 5% of patients needed more extensive mini-PCNL treatment. The effectiveness of UM-PCNL as a safe, minimally invasive alternative for stones up to 20 mm is demonstrated by the mean operative time of 39.7 ± 15.4 min and the hospital stay of 22.3 ± 2.2 h [35].

Beyond single-arm outcome studies, UM-PCNL has been compared with flexible ureteroscopy (fURS) for medium-sized renal stones to assess clinical outcomes and cost-effectiveness. No significant differences between UM-PCNL and fURS were found with respect to operative time (121 min vs. 102 min), length of hospital stay (2.3 days vs. 2.0 days), stone-free rate (84% vs. 87%), and complication rates (~7% in both groups). However, UM-PCNL was more cost-effective than fURS, costing 656 euros in total instead of 1160 euros for each procedure. These results affirm UM-PCNL as a clinically effective and economically favorable minimally invasive treatment for medium-sized urinary stones [36].

While UM-PCNL was initially designed for small to moderate renal stones, a prospective matched study evaluated UM-PCNL in adults with renal stones up to 35 mm, assessing its role beyond the traditional small stone cases. Intra- and postoperative outcomes showed no significant differences except for a longer mean operative time of 130.1 min and a longer hospital stay of 91.5 h compared to flexible ureteroscopy (fURS). Complications of Clavien grades II and III were present in 16% of UM-PCNL patients compared to 4% in fURS patients. Even though UM-PCNL is generally considered more invasive than fURS, the Freiburg Index of Patient Satisfaction was similar in both groups (UM-PCNL 1.73 and fURS 1.67). The findings support UM-PCNL suitability for larger renal stones when a smaller tract size is preferred [37].

#### 3.4.3. Micro-PCNL

Compared to larger, more compact methods like UM-PCNL, micro-PCNL is a further development of miniaturized percutaneous nephrolithotomy, incorporating needle-sized (<5 Fr) renal access with the goal of minimizing parenchymal trauma and morbidity.

Comparative studies have shown stone-free outcomes similar to other miniaturized techniques and associated with lower hemoglobin drop and shorter hospital stays [38,39,40].

The stone-free rate was 90% in a study involving 20 patients with renal stones of mean size 13 ± 3 mm who had a micro-PCNL procedure. Two of the patients had significant residual fragments (≥4 mm). The mean operation time was 107.5 ± 37 min, and fluoroscopy time was 45 ± 40 s. Postoperative outcomes were favorable, with a mean hemoglobin drop of 1.2 ± 0.9 g/dL, with one patient requiring transfusion [41]. In a comparative study of micro-PCNL with retrograde intrarenal surgery (RIRS) in 111 procedures, micro-PCNL was associated with a significantly higher stone-free rate (80.9% for micro-PCNL vs. 66.6% for RIRS) and was more cost-effective ($831.6 ± 79.5 for micro-PCNL compared to $917.1 ± 73.6 for RIRS). In both studies, micro-PCNL resulted in high stone-free rates, short hospitalizations, and favorable cost profiles. However, operative times were longer and there was a minor risk of bleeding. However, micro-PCNL is technically a demanding option, particularly for small renal stones [42].

A randomized controlled trial compared retrograde intrarenal surgery (RIRS) and micro-PCNL in the treatment of renal calculi under 1.5 cm in size. The results showed a similar high stone clearance of 97.1% for micro-PCNL and 94.1% for RIRS. However, double-J stents were needed for a smaller proportion (20%) of micro-PCNL cases compared to 62.8% of RIRS cases, and micro-PCNL was linked to a much larger drop in hemoglobin (0.96 for micro-PCNL vs. 0.56 g/dL for RIRS). On the other hand, micro-PCNL was associated with heightened postoperative pain and higher analgesic needs. Both groups had comparable rates of hospital stays and postoperative febrile episodes (Modified Clavien–Dindo grade I). Overall, the complications were low, and none of the patients required blood transfusion. The results affirm the safety and efficacy micro-PCNL for small renal stones, although with a slightly higher risk of bleeding and postoperative discomfort [43].

A study involving 116 patients with lower calyceal stones of sizes ≤ 2 cm randomly divided participants into two equal groups. The first group was treated with the “all-seeing needle” optical puncture system micro-PCNL, which provides direct visualization of the collecting system through the needle during access. The second group received treatment with flexible ureteroscopy (fURS). The stone-free rate was similar in both groups (84.5% micro-PCNL vs. 79.3% fURS), whereas the mean operative time was significantly longer in the fURS group. No significant differences were observed regarding hemoglobin drop, complications, and length of hospital stay. With the benefit of a shorter operating time than fURS, these results validate the safety and effectiveness of micro-PCNL for treating lower calyceal stones of ≤2 cm [44].

A systematic review, covering the period from 1990 to 2017, supports the effectiveness and safety of micro-PCNL in adult patients, as a suitable option for certain cases. Micro-PCNL was characterized by a stone-free rate (SFR) of 89%, and complication rate of 15.2%, including complications of Clavien I (44%), Clavien II (28%), and Clavien III (28%) grades [45]. Further evidence is provided by a retrospective multicenter case series in which 31 consecutive micro-PCNL procedures were performed for nephrolithiasis (<2.5 cm). The mean stone size was 19 mm ± 11 mm, with localization primarily in the lower calyx (68%). Technical success was observed in 78% of the cases—42% were completely stone-free, and 36% had residual fragments smaller than 3 mm that did not require any additional intervention. The mean operative time of 83 ± 35 min gradually decreased within a short period, indicating a relatively short learning profile. The 29% complication rate mainly included fever in 26% (Clavien II) and a single incidence of renal colic requiring a double-J stent (Clavien III). Both patients with and without urinary diversion had similar outcomes, suggesting a possible advantage in anatomically challenging cases, where retrograde ureteroscopy may be limited [45]. Combining micro-PCNL with flexible ureteroscopic lithotomy was highly effective for refractory lower calyceal stones of 1 or 2 cm. At the third month, the stone-free rate was 96.4% with a significant reduction in VAS pain scores after surgery and minimal complications. This demonstrates the effectiveness of hybrid approaches, which can be used in complex situations where a single modality might not be helpful [46].

## 4. Comparison of PCNL Techniques

### 4.1. Stone-Free Rates

The primary efficacy endpoint for all PCNL platforms is the stone-free rate (SFR); however, it is difficult to make direct comparisons among standard, mini-, ultra-mini-, and micro-PCNL due to varying definitions of SFR. For example, there are differences in imaging modalities, variations in follow-up time points, and distinctions between having no fragments versus having clinically insignificant residual fragments. It is particularly complicated when comparing miniaturized techniques with standard PCNL, where active fragment extraction is often more viable. A systematic review focused on tract sizes across miniaturized PCNL. A wide variation in access caliber was revealed; however, stone-free outcomes were broadly comparable to standard PCNL. It was also noted that cross-technique interpretations were limited by differences in study designs and definitions of outcomes [47].

When standard PCNL is compared with mini-PCNL, stone-free rates (SFRs) are similar, with differences emerging mainly in specific subgroups and depending on comparator tract size. A meta-analysis of randomized control trials established no significant difference in SFR between mini-PCNL and standard PCNL in stones larger than 2 cm, indicating that mini-PCNL can maintain clearance even in larger stone burdens, within the constraints of trial inclusion criteria and surgeon experience.

This body of evidence indicates that standard PCNL cannot be viewed as a single comparator, especially when the control arm employs intermediate tracts. The expected SFR advantage of standard PCNL may be more apparent than when compared against 30 Fr tracts because the technical gap in extraction capacity is narrower, and the mini-PCNL working channel becomes a more prominent limiting factor. Practically, this means SFR comparisons should be interpreted in the context of local standard practice (24 Fr vs. 30 Fr), and authors should avoid overgeneralizing results obtained against one standard caliber to all conventional PCNL [48].

Stone-free rates (SFR) in ultra-mini percutaneous nephrolithotomy (UMP) have less scientific support and are more reliant on stone size. Small-to-medium renal stones are typically treated with UMP, and aggregate results indicate a good clearance rate in that size range. UMP demonstrated a stone-free rate (SFR) of nearly 80% for a mean stone size of 13.9 mm and an SFR of 88.3% for a mean stone size of 18.6 mm [44]. In a randomized control trial comparison of UMP versus retrograde intrarenal surgery (RIRS), the UMP arm achieved a higher 1-month SFR than RIRS. Although this is not a PCNL-to-PCNL comparison, it supports the efficacy of UMP for appropriately selected stone sizes and indirectly shows it as a percutaneous bridge between mini-PCNL and flexible ureteroscopy [49]. A key threat to the robustness of the results concerns the way the stone-free rate is defined and assessed. For example, SFR tends to fall when computed tomography (CT) is applied and residual dust fragments become more visible.

For micro-PCNL, the accuracy of reported stone-free rates (SFRs) depends on the quality of the imaging. A systematic review and meta-analysis comparing microperc and miniperc found nearly identical pooled SFRs (microperc 87.29% vs. miniperc 86.59%), suggesting that extreme tract downsizing does not impact efficacy [38]. However, equivalence in SFR at the aggregate level should not be misinterpreted as equivalence in procedural mechanisms. Microperc often relies on complete laser fragmentation with minimal extraction. This can shift clearance toward spontaneous passage, potentially prolonging the time required to achieve true stone-free status even when early postoperative imaging appears promising. Immediate or early postoperative imaging may underestimate later clearance after dust passage, whereas delayed imaging may overestimate procedural efficacy if further treatments or spontaneous passage occur.

Across the full spectrum, the most robust comparative conclusion is that SFR differences are generally smaller than expected when techniques are applied within their intended indications. The quality of evidence is often limited by inconsistent SFR definitions and heterogeneous imaging. Standard PCNL remains the reference for very large and complex stones because it allows for large-bore access, enabling rapid fragment evacuation and flexible nephroscopy. On the other hand, miniaturized techniques achieve competitive stone-free rates (SFR) for selected stones by sacrificing extraction capacity in favor of reduced invasiveness and requiring a more strategic approach to fragmentation. A critical consideration for comparative analyses is whether the observed SFRs reflect the proper application of procedures or result from inappropriate methods, such as using micro- or ultra-mini percutaneous nephrolithotomy for stone burdens that are better suited for mini- or standard PCNL. Differences in adjunctive flexible nephroscopy, or systematic variation in imaging sensitivity, may affect generalizability [9,38,47]. Because single-session clearance is the most clinically meaningful measure of efficacy when headline stone-free rates appear similar across techniques, it is imperative to account for stone burden and complexity, apply sensitive and standardized imaging, and report stone-free status in conjunction with the need for secondary procedures for reliable comparisons among studies.

### 4.2. Operative Time

Operative time is an indicator that reflects stone complexity, access strategy, fragmentation and evacuation efficiency, and team familiarity with a given surgical circumstance. Across the PCNL spectrum, smaller tracts can reduce the time spent on access-related steps such as dilation and sheath placement. However, an increase in the time spent on intrarenal fragmentation and clearance is often observed because limited outflow and smaller working channels reduce passive fragment egress and constrain extraction tools. Consequently, comparisons of operative time are highly sensitive to procedural workflows such as “fragment-and-extract” versus “dust-and-washout,” whether suction is available, and whether a unit’s standard PCNL is closer to 24 Fr or 30 Fr [9,21].

When comparing mini-PCNL with standard PCNL, randomized evidence consistently shows a modest time penalty for miniaturized tracts despite the magnitude being variable and often heterogeneous. In a meta-analysis of randomized trials that compared miniaturized PCNL with standard PCNL, operative time was significantly longer in mini-PCNL (mean difference = 10.98 min). Variations were attributed to differences in surgeon experiences and institutional practice patterns [21]. Operative time varied and favored standard PCNL; however, even when it was statistically detectable, it was clinically modest [9]. Subgroup analysis in meta-analysis of randomized control trials demonstrated that in stones ≥ 2 cm, mini-PCNL carried a longer operative time (mean difference = 12.26 min). The results are consistently show that larger burdens require more fragmentation to enable clearance through smaller channels. This suggests that miniaturization does not slow PCNL, but rather, it redistributes time from extraction to fragmentation and evacuation steps. The time penalty is proportional to the stone burden and the requirement for careful clearance through smaller-caliber access.

Additional nuance is provided by research centering larger stones. For renal stones >2 cm, a meta-analysis of randomized control trials comparing mini-PCNL and standard PCNL identified shorter operative time in the standard PCNL arm (weighted mean difference = 8.23 min). Efficacy endpoints were broadly similar, indicating that operative time becomes a meaningful trade-off when larger stones are treated through reduced-caliber tracts [48]. This phenomenon has practical implications for technique allocation. In high-volume settings where theater efficiency is constrained, standard PCNL may retain an advantage in time-to-clearance for large burdens, whereas mini-PCNL can be selectively used when reducing access-related morbidity is the focus and the expected operative time is acceptable.

For UMP, comparative operative-time data are more limited and strongly influenced by the stone-size range typical for UMP. In a randomized clinical trial of 10–20 mm renal upper ureteric stones comparing UMP with standard PCNL, there was no significant difference in operation time between groups, despite significant differences in recovery endpoints [26]. This reflects how in smaller stones, where total fragment volume is lower and extraction demands are modest, the expected miniaturization time penalty may not manifest, particularly when the technique is optimized. Conversely, single-arm UMP series often report relatively short mean operative times in carefully selected stone sizes (mean operative time 39.7 ± 15.4 min in an early UMP series). This suggests that both case selection and platform maturity may influence time outcomes [35].

For micro-PCNL, the operative time is distinct because the platform is designed around single-step access with a very small tract and a fragmentation pattern. Comparative analysis suggests that extreme downsizing does not necessarily impose a time penalty relative to miniperc in the populations studied. A meta-analysis comparing microperc with miniperc found no significant difference in operative time (weighted mean difference = 5.76, *p* = 0.62), implying that the access simplification and reduced tract management of microperc can offset the increased reliance on fine fragmentation in selected stones [38]. However, operative time in microperc is particularly sensitive to laser efficiency, visibility, and the learning curve. Case reports emphasize that shortened operating times are achievable, but platform familiarity is imperative to avoid prolonged fragmentation and repeated adjustments [45].

Finally, operative time should be interpreted not only as an efficiency metric but also as a safety signal, because longer operations can reflect technical difficulty and can correlate with worse postoperative courses. In the global CROES study with 5724 PCNL patients, longer operative time was associated with a higher risk of more severe postoperative complications, supporting the view that minimizing unnecessary operative prolongation is clinically relevant across PCNL techniques [16]. Overall, the existing evidence suggests that miniaturization generally results in a small to moderate increase in operative time for mini-PCNL, particularly as the stone burden increases. While UMP may show time equivalence in smaller stones, microperc may achieve comparable operative times to miniperc in selected cases. The most clinically meaningful comparisons are those that stratify by stone burden and complexity and report operative time alongside workflow determinants. These variables can account for between-study variability more reliably than tract size alone [21,26,38].

### 4.3. Complications and Safety Profile

Through the PCNL spectrum, safety relates to bleeding-related morbidity, infectious complications, and access or collecting-system injury, because these are the domains most plausibly influenced by tract caliber and intrarenal fluid dynamics. Across studies, comparisons are complicated by inconsistent reporting of complications due to non-standardized use of the Clavien classification system, variations in stone complexity, and differences in the standard sizes of PCNL sheaths (24 Fr vs. 30 Fr) [47,48,50].

Bleeding and transfusion are the most consistently tract-linked outcomes. A meta-analysis comparing mini-PCNL with standard PCNL reported a smaller hemoglobin drop (mean difference = 4.67 g/L) and markedly lower transfusion risk (Odds ratio = 0.18) for mini-PCNL [51]. A later meta-analysis also found fewer transfusions with mini-PCNL (Odds ratio = 0.36) and a smaller hemoglobin decrease (mean difference = 0.68). Overall, adverse-event profiles were broadly similar, suggesting that miniaturization can reduce access-related hemorrhagic morbidity but does not uniformly lower all complication types [22]. The apparent safety gap is significantly altered by the tract size within the typical PCNL. A systematic review which focused on renal stones larger than 2 cm showed that hemoglobin drop and transfusion were more common when the standard arm used 30 Fr access, with transfusion risk substantially lower in the mini-PCNL arm [43]. The assertion that standard PCNL morbidity is not uniform indicates variability in outcomes. Furthermore, it suggests that the bleeding advantages associated with mini-PCNL may be particularly significant when compared to the 30 Fr practice. However, these advantages may diminish when comparing mini-PCNL to 24 Fr techniques.

The best evidence for the safety advantages of ultra-mini PCNL arises from randomized studies that focus on stone sizes smaller than 20 mm. In a randomized control trial comparing ultra-mini PCNL using a 16 Fr sheath with standard PCNL using a 30 Fr sheath, postoperative hemoglobin drop was 1.65 g/dL vs. 3.13 g/dL, and transfusion rates were 5.71% vs. 11.4% in favor of ultra-mini PCNL. Perioperative complications were infrequent and included low fever counts and isolated bleeding events [26]. The results indicate that there are advantages for treating smaller stone burdens, but these benefits may not apply to large staghorn stones. Prolonged lithotripsy performed through very small access points may affect pressure, visibility, and the dynamics of infection risk.

A meta-analysis comparing microperc with miniperc showed that microperc had a lower hemoglobin drop but higher renal colic requiring double-J stent insertion (odds ratio = 3.49), while urinary tract infection rates did not differ significantly [38]. This pattern highlights how extreme tract downsizing may reduce parenchymal trauma yet shift postoperative events toward colic or obstructive symptoms related to fragment passage strategies and drainage choices. In summary, a reduction in invasiveness does not guarantee the absence of morbidity in clinical practice.

Infectious complications, including fever, urinary tract infections (UTIs), and sepsis, are not improved as consistently by miniaturization compared to bleeding. A meta-analysis showed that postoperative fever did not differ between mini and standard PCNL, despite transfusion benefits, implying that infection risk is driven by factors beyond tract size alone [51]. This trend is also reflected in broader meta-analytic work emphasizing that, while mini-PCNL may improve blood-loss metrics, differences in fever and other adverse events can be statistically non-significant and highly heterogeneous across trials [21]. Miniaturization provides its most reliable safety advantage in bleeding/transfusion, whereas infection outcomes are more contingent on a case-by-case basis and technique application (pressure control, operative duration, and drainage strategy) as opposed to solely tract caliber.

From a fluid-dynamics perspective, intrarenal (intrapelvic) pressure rises when irrigation inflow exceeds effective outflow. Miniaturized tracts may limit drainage capacity because smaller working channels and access sheaths increase flow resistance. Transient occlusion by instruments or stone fragments can further impair egress. Elevated intrarenal pressure promotes pyelovenous and pyelolymphatic backflow, creating a physiological pathway for bacterial movement and endotoxin absorption. Elevated pressure is associated with postoperative infectious complications, particularly in cases of infected systems, obstructed kidneys, prolonged procedures, or high-irrigation workflows. This highlights the significance of managing pressure as a flexible safety measure in miniaturized PCNL [31,33,34].

### 4.4. Hospital Stay and Postoperative Recovery

Hospital stay and postoperative recovery are clinically meaningful end points because they integrate multiple downstream effects of the chosen PCNL platform, including access-related trauma, bleeding, drainage strategy (nephrostomy vs. tubeless), postoperative pain, and early complications. The length of a hospital stay is significantly influenced by the healthcare system in place, including factors such as enhanced recovery pathways, discharge criteria, and outpatient follow-up.

Comparative studies of mini-PCNL versus standard PCNL consistently demonstrate that miniaturization leads to shorter hospital stays. This effect may be partially influenced by practice patterns, such as the increased use of tubeless strategies. A recent meta-analysis comparing mini-PCNL and standard PCNL indicated that the mini-PCNL group experienced shorter hospital stays and a higher rate of postoperative tubeless outcomes, while no significant difference in VAS postoperative pain was observed [52]. The lack of a consistent VAS difference implies that discharge timing could be affected as much by tube-related recovery and institutional discharge protocols as by pain alleviation alone. Although the observation that less invasive techniques contribute to reduced hospital stays is well-supported at the population level, it should not be solely attributed to decreased pain unless pain and analgesic outcomes are analyzed within the same cohort.

Drainage strategy is a major determinant of recovery irrespective of tract size, and it can impact comparisons that do not standardize exit approaches. A meta-analysis of randomized control trials comparing tubeless PCNL with no nephrostomy or ureteric stent with standard PCNL demonstrated a significantly shorter length of hospital stay for tubeless PCNL (mean difference = 1.10 days). The analysis revealed no significant differences in pain scores or the number of patients needing pain medication. This suggests that the shorter length of hospital stay may be attributed to earlier mobilization and easier discharge when nephrostomy-related care—such as tube discomfort, leakage, and the logistics of tube removal—is avoided, rather than to substantial variations in early pain scores [53]. A Cochrane review evaluating tubed, tubeless, and completely tubeless PCNL similarly concluded that tubeless approaches may reduce length of stay compared with standard tubed PCNL, reinforcing the central role of exit strategy in recovery outcomes across percutaneous platforms [54].

For ultra-mini PCNL, evidence suggests a distinct recovery advantage for smaller stones attributed to reduced access trauma and streamlined postoperative management. In a randomized clinical trial with 10–20 mm upper renal ureteric stones, ultra-mini PCNL was associated with a significantly shorter duration of hospitalization and lower postoperative VAS pain scores than standard PCNL, with no significant differences in operative time or stone-free rate. While this study indicates a recovery benefit for ultra-mini PCNL, its generalizability may be limited to smaller stone sizes. This is because ultra-mini PCNL is generally not utilized for large or complex stones, where extended intrarenal manipulation could potentially diminish the recovery advantages [26].

For micro-PCNL, the literature suggests that the length of hospital stay may be similar to miniperc, even if specific postoperative experiences differ. A meta-analysis comparing microperc and miniperc reported no statistically significant difference in length of hospital stay [38]. The results are consistent with the concept that recovery can be limited by factors other than tract caliber, such as postoperative colic, stenting decisions, residual fragment passage, and institutional discharge protocols.

Across PCNL techniques, there are three comparative trends. First, shorter hospital stays are typically linked to miniaturization (mini- and ultra-mini), although the effect varies and may be partially mediated by higher rates of tubeless or less taxing drainage techniques [54,55]. Second, meta-analyses do not consistently support the pain advantages of mini-PCNL, while ultra-mini-PCNL demonstrates reduced postoperative pain. This indicates that the observed benefits in pain management may vary depending on the specific clinical indications and the platform used for the procedure. Third, drainage strategy independently shortens the length of hospital stay across PCNL approaches. Thus, recovery comparisons are most reliable when tract size and exit strategy are either standardized or analyzed as interacting factors rather than isolated variables [53,54].

## 5. Summary of Key Aspects of the Discussed PCNL Techniques

Table 1 outlines the definitions of access and tracts, representative instruments, energy sources, methods for managing fragments, and variations in procedures across the PCNL techniques covered in this review article.

## 6. Emerging Technologies and Future Developments in Miniaturized PCNL

Emerging technologies in miniaturized PCNL range from tools already used in clinical practice to investigational platforms still undergoing early validation. Ongoing miniaturization of PCNL has been driven by advances in optics, access sheaths, and laser lithotripsy, designed to reduce bleeding and hospital stay while maintaining satisfactory SFR. The major limitations include lower stone-free rates (SFRs) for stone sizes greater than 2 cm, longer operative time, and an increase in intrarenal pressure, potentially raising the risk of infection. This emphasizes the need for improvements in irrigation and pressure control techniques. Recent reviews suggest the 18 Fr mini-PCNL is the most suitable option for stones up to 25 mm; however, retrograde intrarenal surgery (RIRS) remains the treatment of choice for stones smaller than 10 mm [13].

The continuous technological refinements gave birth to the development of very small-caliber systems focused on reducing morbidity while preserving SFR. A major expert review shows advances such as endoscopic-guided real access combined with antegrade and retrograde approaches may reduce morbidity, improving precision [55]. The topic of intrarenal pressure has become increasingly important in modern endourology. Recent systematic reviews concluded that mini- and micro-PCNL can accomplish a lower intrarenal pressure (IRP) via optimized irrigation and vacuum-assisted sheaths. Continuous measurements of pressure combined with intelligent pressure regulators are important to reduce the risk of infectious complications. The development and adoption of more advanced intraoperative pressure management tools and automated pressure control systems offer a potential technological advance that may further improve safety in miniaturized PCNL [60].

Emerging engineering studies are shifting miniaturized PCNL beyond procedural techniques by optimizing the individual components. Benchtop evaluations are underway to assess the impact of different lithotrite technologies on stone fragmentation. Comparative bench testing of multiple mini-PCNL lithotrites utilizing certain mechanical devices, such as the Trilogy 1.9 mm probe, displayed a greater stone mass clearance rate as opposed to traditional laser fibers in an artificial model. This opens avenues for optimizing energy delivery and device design in miniature access sheaths [61].

Novel engineering technologies such as the 120-watt Moses Pulse holmium laser system with anti-retropulsion have been designed to control intrarenal pressure (IRP) closer to physiological levels (<20 mm Hg) as opposed to current uncontrolled systems where pressures may exceed 30–40 mm Hg. It has produced favorable results in the preliminary stages, yielding a 92% stone-free rate with a median operative time of 68 min, a mean laser time of 24 min, and a mean energy delivery of 22.6 kJ without any major complications. Other comparisons between various mini-PCNL lithotrites show that advanced mechanical devices like the Trilogy 1.9 mm lithotrite could clear stones substantially faster than traditional fibers [59]. The findings indicate that improvements in miniaturized PCNL should focus not only on decreasing tract size but also on enhancing the clinical effectiveness of mini-PCNL systems [59,61].

Contemporary technology has been engineered to address not just intrarenal pressure (IRP) but also other important limiting factors of extreme miniaturization, such as fragment evacuation, visualization, and optics. Recent evaluations of vacuum-assisted sheaths incorporating high-flow irrigation and vacuum fluid dynamics vs. mini-PCNL report a significant reduction in mean operative time (95 min vs. 146 min) and shortening of length of hospital stay (1.7 ± 1.9 days vs. 2.7 ± 1.5 days) [56]. Mini-PCNL, performed with vacuum-assisted sheaths, has been engineered to provide continuous high-flow irrigation and suction-enabled evacuation of fragments, showcasing a step toward device design to reduce the limitations of miniaturization [57]. In complex cases, like obstructive pyonephrosis, it has been used with no increase in morbidity and a significant reduction in operative time (60.6 ± 7.7 min vs. 82.2 ± 14.0 min) and postoperative fever rates (8% vs. 32%) [58].

Novel navigation systems and 3D imaging technology may improve PCNL visualization and access precision beyond the current traditional 2D fluoroscopic or ultrasound methods. The result allows detailed anatomical mapping and enhances the intraoperative guidance. It is still in its early stages of development and requires further clinical validation; however, it could facilitate training, safer access, and more accurate puncture [62].

Multimodal fusion imaging combining computed tomography and ultrasound has been shown to enhance first-attempt puncture success to 95% compared to standard ultrasound success of 80% in vitro and in vivo. The ultrasound screening time decreased from 3.37 ± 0.51 min to 2.60 ± 0.33 min. As miniaturization of PCNL evolves further, access accuracy becomes compromised, and multimodal fusion can enable precise, first-attempt calyceal puncture, allowing a safer and reproducible method [63].

Modern robotics has revolutionized PCNL, resulting in improved surgical outcomes while enhancing surgeons’ capabilities. Early real-time navigation systems, such as SonixGPS, showed successful procedures with high stone-free rates (92%) without major complications [64]. Recent advances in 5G technology in tele-assistance surgery have enabled the development of Automated Needle Targeting with X-ray (ANT-X), which allows image registration and closed-loop feedback to automatically align the targeted needle to a zone in the calyx. Fifteen patients had successful PCNL with a 78.6% first-renal puncture success rate, despite a 177 ms delay for more than 5800 km. Navigation systems integrated with robotics reduce technical errors and improve needle tract accuracy while allowing immediate feedback to the surgeon. Robotic assistance under fluoroscopic guidance has been more commonly used for intraoperative PCNL maneuvers than X-ray-guided renal access. ANT-X uses AI-based real-time analysis to calculate and precisely locate the targeted calyx [65].

Robotic-assisted fluoroscopic guidance for PCNL has undergone randomized controlled trials in animal and human studies, reducing the number of needle punctures by 0.73 on multivariate analysis [66]. New navigation systems using real-time electromagnetic (EM) sensors have been shown to offer radiation-free, safe, and accurate renal puncture with success in the first attempt in all the patients involved [67].

Despite the technological advances in PCNL, most platforms remain in their early clinical use. Various factors, such as cost, learning curves, and limited high-quality data, restrict its widespread adoption, especially in certain areas with limited access to specialized training programs. This situation underscores the need for further validation before these technologies can be integrated into the practice of miniaturized PCNL [68].

## 7. Conclusions

PCNL has evolved from highly effective but invasive standard techniques into a range of miniaturized techniques with its primary focus on maintaining good stone clearance while simultaneously reducing patient morbidity. The current limitations of standard PCNL, including bleeding risk, postoperative pain, extended hospital stays, and other stone-related burdens, have prompted the development of diverse miniaturization techniques, each offering its own advantages. Clinical evidence has revealed that miniaturization techniques can achieve comparable results to standard PCNL in terms of stone clearance rate and perioperative safety, together with faster recovery in appropriate patients.

There are also benefits in bleeding reduction and postoperative recovery. However, a definitive consensus has not yet been established, largely due to heterogeneity in study designs, SFR definitions, imaging thresholds, and variability in access caliber and platforms across studies.

As miniaturization has advanced, important trade-offs have become apparent, including longer operative times, increased technical complexity, and greater sensitivity to IRP management, fragment evacuation efficiency, and visualization. The encountered problems have become a catalyst for innovations and advances in engineering solutions, such as suction-integrated sheaths, multimodal image fusion guidance, navigation systems, and robotic assistance. The full potential of these advancements, which aim to improve the PCNL process in terms of accuracy, safety, and consistency, is not yet fully realized.

The future of miniaturization is not just dependent on the size of the tracts. There is a need for high-quality, multicenter randomized trials using standardized outcome assessments of stone-free rates, infectious endpoints, and pressure-related safety metrics. This will allow meaningful and reliable cross-platform comparisons and conclusions. In addition to technical viability, practical factors like procedure cost, equipment availability, institutional resources, and surgeon learning curves must be taken into account for the successful clinical use of miniaturized PCNL techniques. These factors are likely to play an important role in determining whether newer technologies translate into clinical benefit beyond specialized centers. Accordingly, the future of PCNL is not just dependent on reduction in tract size alone but rather on integrated technological and procedural strategies capable of maintaining efficacy, safety, reproducibility, and accessibility, ensuring durable benefits in routine clinical practice.

## Figures and Tables

**Table 1:** Key aspects of the discussed PCNL techniques.

Key Aspects	Standard PCNL	Mini-PCNL	Ultra-Mini PCNL	Micro-PCNL	Suction or Vacuum PCNL
Access and tract	Standard-caliber percutaneous access exceeding 22 Fr [47].	Access tracts between 14 and 22 Fr [23,27,28,47]	Access sheaths typically around 11 Fr to 16 Fr [24,26,35]	Single-step access; Tract sizes around 4.85 Fr [25]	Usually mini-range; Vacuum-assisted access [33,34]
Instrumentation	Rigid nephroscopy, standard access sheaths, conventional multi-step access, dilation, lithotripsy, and fragment extraction [14,15,16,17]	Smaller peel-away sheaths and miniaturized nephroscopes that are being further refined. [23,27,28,29]	Small-caliber metal sheaths with mini-nephroscopes; ureteroscopes with reduced-caliber sheaths, ultra-mini optical systems [24,26,35]	Micro-optic systems-all-seeing needle platform [25,42,43]	Sheaths such as ClearPetra and other aspiration-enabled systems [33,34,56,57,58]
Energy sources	Intracorporeal lithotripsy techniques vary by center and study, and energy selection is not standardized [15,16,17,20]	Laser lithotripsy, with energy selection tailored to fragmentation through a reduced working channel [27,29]	Holmium YAG laser lithotripsy; high-power laser systems discussed in emerging technology [24,59]	Laser-based lithotripsy with energy delivery optimized for complete intrarenal fragmentation through a very small working channel [25,43]	Energy source depends on the underlying miniaturized PCNL. Suction functioning as a clearance and pressure- control adjunct [33,34]
Fragment management approach	Active fragment extraction facilitated by large-bore access and efficient outflow [15,16,17]	Greater reliance on fine fragmentation and controlled clearance [27,28,29]	Fragmentation -focused; limited extraction; dusting and clearance through irrigation and small-caliber access systems [24,35]	Complete fragmentation with minimal or no active extraction; reliance on fragment passage and adjunct drainage [25,38,42]	Continuous suction- assisted evacuation of fragments combined with irrigation [33,34,56,57,58]
Procedural variations	Across all variants of percutaneous nephrolithotomy (PCNL), the use of a nephrostomy tube is optional rather than mandatory. In miniaturized PCNL, where the tract is smaller, the procedure is more likely to be tubeless. This is because the risks of bleeding and urinary extravasation are lower, and the tract itself is less traumatic.				
	Commonly tubed, but in uncomplicated cases maybe tubeless [53,54]	More frequently, tubeless with JJ stent; Mini-nephrostomy in specific cases [30,31,32,33,34,53,54]	Typically, tubeless with JJ stent; Totally tubeless in uncomplicated cases [24,35,36,37,53,54]	Tubeless by design with JJ-stent frequently left; Totally tubeless in uncomplicated cases [25,38,42,43,46]	More often tubeless, but tubed drainage is also applied in more complex cases. [33,34,56,57,58]

## Data Availability

No new data were created or analyzed in this study.

## Abbreviations

The following abbreviations are used in this manuscript:

ADC	Apparent diffusion coefficient
AI	Artificial intelligence
ASA	American Society of Anesthesiologists (physical status classification)
CROES	Clinical Research Office of the Endourological Society
CT	Computed tomography
ECIRS	Endoscopic combined intrarenal surgery
IRP	Intrarenal pressure
IPP	Intrapelvic pressure
LOS	Length of stay
MD	Mean difference
MDCT	Multidetector computed tomography
MPCNL	Mini-percutaneous nephrolithotomy (mini-PCNL)
OR	Odds ratio
PCNL	Percutaneous nephrolithotomy
RCT	Randomized controlled trial
RIRS	Retrograde intrarenal surgery
RR	Risk ratio
SFR	Stone-free rate
SWL	Shock wave lithotripsy
UM-PCNL	Ultra-mini percutaneous nephrolithotomy
UTI	Urinary tract infection
VAS	Visual analogue scale
WMD	Weighted mean difference
YAG	Yttrium aluminum garnet (holmium:YAG laser)
